# Pregnant Women and Vaccine Safety in Uganda: Knowledge, Barriers, and Opportunities for Engagement

**DOI:** 10.3390/vaccines13121210

**Published:** 2025-11-30

**Authors:** Victoria Prudence Nambasa, Robinah Komuhendo, Allan Serwanga, Dan Kajungu, Birgit C. P. Koch, Beate Kampmann, Kirsty Le Doare, Agnes Ssali

**Affiliations:** 1 Erasmus MC, University Medical Centre, Department of Hospital Pharmacy, Postbox 2040, 3000 CA Rotterdam, The Netherlands; b.koch@erasmusmc.nl; 2 Department of Community Health and Behavioral Sciences, Makerere University School of Public Health, Kampala P.O. Box 7072, Uganda; komuhendorobina@yahoo.co.uk; 3 National Drug Authority, Kampala P.O. Box 23096, Uganda; aserwanga@nda.or.ug; 4 Makerere University Centre for Health and Population Research (MUCHAP), Kampala P.O. Box 7062, Uganda; dkajungu@muchap.mak.ac.ug; 5 Division of Epidemiology and Biostatistics, Department of Global Health, Stellenbosch University, Private Bag X1, Stellenbosch 7599, South Africa; 6 Charité Centre for Global Health, Charité Universitätsmedizin, 10117 Berlin, Germany; beate.kampmann@charite.de; 7 Faculty of Infectious & Tropical Diseases, London School of Hygiene & Tropical Medicine, London WC1E 7HT, UK; 8 Makerere University-Johns Hopkins University Research Collaboration, Kampala P.O. Box 23491, Uganda; kiledoar@sgul.ac.uk (K.L.D.); agnes.ssali@mrcuganda.org (A.S.); 9 Centre for Neonatal and Paediatric Infection, St George’s, University of London, London SW17 0RE, UK; 10 Medical Research Council/Uganda Virus Research Institute & LSHTM Uganda Research Unit, Entebbe P.O. Box 49, Uganda

**Keywords:** vaccines, pregnant women, adverse events following immunization, safety surveillance, pharmacovigilance

## Abstract

**Background/Objectives**: New vaccines designed to combat infections such as Group B Streptococcus and respiratory syncytial virus will soon be accessible in Africa. While outbreak response vaccines are given to pregnant women, safety data for maternal vaccines in low- and middle-income countries (LMICs) are limited. This study explored Ugandan pregnant women’s knowledge, attitudes, and engagement in adverse event reporting and vaccine decision-making. **Methods**: This nested qualitative study was part of a national gap analysis of pharmacovigilance systems for maternal vaccines. Five Focus Group Discussions (FGDs), each involving eight participants, were held with pregnant and or breastfeeding women at four healthcare facilities and one research center. The data collected from these discussions were analyzed thematically using a manifest content analysis, conducted in Atlas.ti software version 9 for qualitative data analysis. **Results**: Women valued maternal vaccines, particularly tetanus, but reported confusion about schedules and hesitancy when informed of potential side effects. Many adverse events were normalized, therefore not reported, and most participants were unaware of national reporting mechanisms beyond informing healthcare providers. Barriers included inadequate information, dismissive or rushed provider interactions and reliance on family, peers, and informal care networks to manage side effects. Women expressed a strong desire to be informed and actively involved in decisions about pregnancy vaccines, including the introduction of new vaccines. **Conclusions**: Strengthening maternal vaccine safety monitoring requires clearer, balanced communication; simplified and well-publicized reporting tools; supportive provider–patient interactions; and integration of community and informal networks. Pregnant women should be engaged as active partners in pharmacovigilance and maternal vaccine introduction to build trust, improve adverse event reporting, and support vaccine uptake.

## 1. Introduction

Vaccination remains one of the most effective public health interventions, preventing morbidity and mortality from infectious diseases, including those that pose risks during pregnancy [1,2]. Despite the clear benefits of vaccination, concerns remain about vaccine safety, particularly potential risks to the mother and fetus, limited data on disease burden and the low public threshold for acceptance of adverse events following immunization (AEFIs) [3,4,5]. Although generally rare, AEFIs must be closely monitored to maintain public confidence and trust in vaccination programs and to safeguard maternal and fetal health [6,7].

Moreover, the anticipated introduction of new vaccines aimed at preventing infectious diseases in pregnant women and newborns, such as Group B Streptococcus (GBS) and Respiratory Syncytial Virus (RSV), in low- and middle-income countries (LMICs) also underscores the importance of supporting existing systems to enable early signal detection, inform benefit–risk assessments, and ensure the safe use of vaccines in pregnancy [7,8,9,10,11]. Empowering patients including pregnant women to participate in AEFIs reporting can enhance pharmacovigilance. Studies suggest that such engagement contributes to improved safety signal detection, supports timely response, and contributes to better quality of care [12,13,14,15]. Pregnant women are particularly important contributors of real-world safety data, especially during pandemic responses or when vaccines are deployed outside clinical trial settings [6,12,16]. Given their typical exclusion from pre-licensure trials, passive surveillance systems serve as critical tools for monitoring vaccine safety in this population [7].

Spontaneous reporting of suspected AEFIs remains the pillar of pharmacovigilance globally, especially in LMICs [17]. While underreporting is a known limitation, the system play a vital role in identifying potential safety concerns [18,19,20]. Patient or consumer reporting is increasingly recognized to be an essential part of the pharmacovigilance system, not only serving as a source of safety information during their healthcare use but also adding value to pharmacovigilance by reporting types of drugs and reactions that differ from those reported by Healthcare professional [12,21,22,23,24,25,26,27].

However, involvement of pregnant women in reporting adverse events following maternal immunization (AEFMI) may be limited due to various barriers, including a lack of awareness of adverse events to report, unclear reporting pathways, sociocultural norms, and health system inefficiencies [28]. Beyond pregnant women’s perspectives on their role in vaccine decision-making to influencing vaccine acceptance, their role in recognizing, interpreting, and reporting AEFIs is poorly understood. Engaging them in discussions before vaccine rollouts can enhance their confidence in immunization programs, address safety concerns, and improve trust in healthcare systems [16].

In Uganda, the national pharmacovigilance system mainly relies on passive surveillance. Generally, the available tools for both the healthcare providers and the public to report suspected adverse events include a toll-free line at the national pharmacovigilance center, an online reporting form, Unstructured Supplementary Service Data (USSD) code, Mobile phone applications [Med Safety APP], and WhatsApp [29]. Despite the availability of these tools, underreporting persists due to various reasons including limited awareness for both healthcare providers and patients [14].

Tetanus Toxoid Td [MV1+] and Tetanus-diphtheria [Td] vaccines are routinely administered in ANC, while COVID-19 vaccines were offered to pregnant women during the pandemic. Additional vaccines like Hepatitis B, Cholera, Meningitis, and Yellow Fever are utilized during vaccination campaigns and disease outbreaks, including for pregnant women when deemed necessary [29].

As Uganda and other LMICs prepare for the introduction of new maternal vaccines, it is essential to understand pregnant women’s perceptions and engagement in the safety monitoring of current vaccines. Evidence concerning their knowledge, beliefs, and willingness to report adverse events following immunization (AEFIs), particularly those pertinent to maternal vaccines such as, pregnancy outcomes, is critical for enhancing pharmacovigilance systems and promoting successful vaccine uptake [30]. This study aimed to evaluate pregnant women’s involvement in AEFI reporting in Uganda, identify barriers to their participation, and examine their perspectives on vaccine-related decision-making in anticipation of the rollout of new maternal vaccines.

## 2. Materials and Methods

### 2.1. Study Design

This was a qualitative sub-study nested within a national gap analysis of pharmacovigilance for vaccines used during pregnancy, conducted in Uganda [29]. A qualitative approach was chosen to facilitate a deeper understanding of the study objective and reality. Focus group discussions (FGDs) were utilized at the same public health facilities and one research center involved in the parent study. The discussion guide was designed to explore pregnant women’s perceptions of vaccination, their knowledge, awareness and experiences with adverse event reporting, and their involvement in decision-making based on a preliminary review of the literature [13,14,15,19,25,31] and informed by professional experience in pharmacovigilance.

### 2.2. Study Site, Population and Sampling

The research was conducted in antenatal care (ANC) clinics across four public health facilities and one affiliated research site, as part of a broader parent study. The facilities involved included a national referral hospital, two regional referral hospitals, and a Health center III, a sub-district facility that provides primary healthcare [29]. The study engaged pregnant women and or breastfeeding mothers attending antenatal care at these selected sites, utilizing purposive sampling methods. Participants included any woman attending ANC during the study period who expressed willingness to participate and provided consent. The recruitment aimed to reach a target of eight participants per FGD.

### 2.3. Eligibility

Pregnant women and or breastfeeding women attending ANC at the time of the study and who had provided written consent to participate were included.

### 2.4. Data Collection

After receiving administrative clearance from the facilities, we collaborated with community leaders and Village Health Teams (VHTs) to identify eligible participants from their assigned ANC services and obtain their consent. We conducted five focus group discussions (FGDs) with pregnant and breastfeeding women who were attending ANC and seeking immunization services at the study sites. The discussions took place over three months (April to June 2023) to accommodate appointment scheduling and site approval. The study aimed to enroll 40 participants in the FGDs, with 8 participants per group. Skilled social workers with pharmacovigilance knowledge (RK, DBN, RNK), supported by research assistants, led the discussions. All discussions were conducted in the local languages, as approved by the ethics committee, and were audio-recorded with permission.

The FGDs were guided by a validated semi-structured interview guide (Supplementary Material S1), tested and refined through a role-play exercise with the research assistants who collected the data. The guide comprised 11 open-ended questions designed to elicit participants’ views and experiences regarding vaccination during pregnancy, and probes were used to ensure depth and clarity in responses. Specifically, the discussions explored six main topics:

1 Knowledge and awareness of vaccines administered during pregnancy (including types, schedules, and benefits);
2 Perceptions of risks and benefits associated with maternal vaccination;
3 Experiences with adverse events following immunization (AEFIs) and perceived severity;
4 Knowledge, sources of information and awareness of AEFI reporting mechanisms
5 Barriers and facilitators to reporting AEFIs;
6 Involvement in decision-making regarding vaccine uptake during pregnancy.

Discussions lasted at least 90 min, were audio-recorded, and participants were compensated in accordance with national ethics requirements.

### 2.5. Data Analysis

We conducted a manifest content analysis [32] to capture the explicit meanings expressed in participants’ statements, while recognizing that some themes required limited interpretive insight guided by the data. All interviews were audio-recorded during the sessions and transcribed verbatim. The transcripts were then imported into Atlas.ti version 9, a qualitative data management software, for coding and analysis. [33]. A pre-developed codebook, developed from a priori concepts identified in the literature and study objectives, guided deductive coding. At the same time, inductive coding enabled the identification of emerging concepts from participants’ narratives, including the community’s role in managing AEFIs, trust in health workers, and women’s sense of ownership over vaccine information. Codes were refined iteratively through constant comparison, with discrepancies resolved through team discussions to ensure reliability. Codes were organized into categories and sub-categories, and subsequently synthesized into broader themes. This dual approach enabled a systematic representation of both predefined and any emerging insights not anticipated a priori.

Two coders independently coded transcripts, and discrepancies were addressed through regular team discussions in which coders reviewed excerpts, refined definitions, and reached consensus. Inter-coder reliability was assessed on 10% of randomly selected transcripts throughout the process by the principal investigator.

## 3. Results

### 3.1. Participant Characteristics

Forty pregnant women participated in five focus group discussions. The majority of the pregnant women were aged 30 or older, with varying levels of education (Appendix A Table A1).

**Figure 1:**
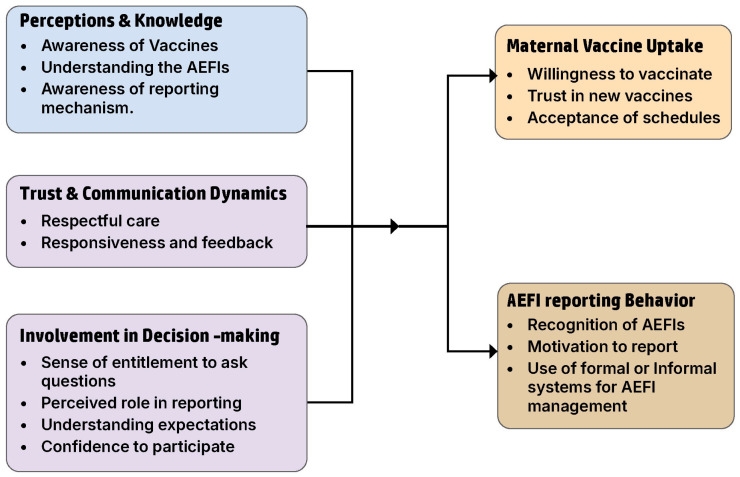
Thematic framework illustrating factors influencing maternal vaccine and AEFI reporting in Uganda.

Key thematic findings are described below, while detailed themes with excerpts are provided in Appendix A Table A2. The themes and their interrelationships are summarized in Figure 1.

### 3.2. Perceptions of Vaccination in Pregnancy

Many women linked maternal vaccines to protective benefits, especially in unsafe delivery conditions that can expose them to infections. Mothers understood the importance of maternal vaccinations, particularly the tetanus vaccine, which they knew well in preventing tetanus infections for themselves and their babies during and after childbirth.

“*The Vaccine is important because it helps both the pregnant woman and the unborn baby not to get the disease*.”

(FGD_Mother_F4 HC III)

“*Vaccines help us in some cases. You can deliver from a dirty place, but if you are vaccinated against tetanus, it helps prevent you from contracting tetanus*.”

(FGD_Mother_F1 RRH)

When asked about their understanding of vaccine schedules and doses, many mothers reported they lacked clear information on when and how often to receive vaccines. Most were uncertain about the number of doses needed and their timing. They mentioned receiving vaccines during ANC visits and relying on healthcare providers for guidance on when to receive their next dose.

“*We do not know the right time when we are supposed to receive the Tetanus injection, but anytime you go is when you can receive it*.”

(FGD_Mother_F1 RRH)

“*Immunization is important, but we don’t know when we should get it or how many times*.”

(FGD_Mother_F2 NRH)

### 3.3. Knowledge and Awareness of AEFI and Reporting Mechanisms

Many pregnant women demonstrated a good understanding of common AEFIs, including arm swelling, pain, and fever. However, none were aware of other potential adverse events that could affect their pregnancy or their babies. Several participants described experiencing these effects themselves after receiving the vaccine. One participant in the FGD reported experiencing a high temperature, weakness, and swelling of the arm. Another mother explained:

“*They immunised me when I was three months pregnant. I developed a fever, and my arm was so swollen that I couldn’t hold anything*.”

(FGD_Mother_F2 NRH)

Women reported that they often did not report adverse effects associated with vaccination, viewing these reactions as normal occurrences and not serious. Common symptoms such as pain at the injection site and mild fever were frequently downplayed, as many were unaware of the reporting mechanisms in place. Additionally, numerous participants reported that healthcare providers reassured them that these side effects were typical and did not require medical attention.

“*Yes, I experienced arm pain for a short period of time. I thought side effects were normal after the injections. I did not know we were supposed to report or tell anyone about it unless it became severe*.”

(FGD_Mother_F1 RRH)

“*They told us that if the arm becomes heavy, we should give it time, and it will return to its normal state*.”

(FGD_Mother_F2 NRH)

Most indicated that their primary understanding of reporting mechanisms involved returning to the health facility and informing their healthcare providers, a practice commonly advised during antenatal care visits. Furthermore, there appeared to be a lack of awareness about alternative channels for reporting these events. One participant articulated this concern succinctly:

“*If you experience any problems after vaccination, you are supposed to return to the health facility and seek advice*.”

(FGD_Mother_F1 RRH)

Another participant reinforced this perspective by stating,

“*They tell you that if you encounter any issues upon returning home, you should come back to the hospital and explain your situation to the health worker so they can assist you in finding a solution*.”

(FGD_Mother_F4 HCIII)

### 3.4. Community as a Source of Guidance in Managing AEFIs (Emergent Theme)

Community members and relatives served as valuable sources of information and alternative forms of support or coping with AEFI, offering advice that both aligned with and contradicted the guidance provided by healthcare professionals for managing AEFIs experienced by women. These findings highlight the role communities play in influencing vaccine use, including AEFI reporting and management.

“*When your arm swells, the people in the village will start advising you to look for herbs like Dodo vegetable (Amaranth leaves), saying that if you smear it on the vaccination site, you will recover*.”

(FGD_Mother_F3 RRH)

“*Once, after being immunised against tetanus, the injection site became itchy, and I started to scratch it. While scratching the itchy patch, I began to feel pain. My sister advised me to put a bottle in the refrigerator and then apply it to the site every morning. I then did so, and the itching and pain reduced*.”

(FGD_Mother_F3 RRH)

### 3.5. Prior Knowledge of Adverse Events and Their Effect on Vaccine Acceptance

Historical harm related to vaccines whether real or perceived affected women’s willingness to go back for further doses, highlighting the importance of the quality of information shared with mothers. Most mothers’ willingness to vaccinate declined when they were aware of possible serious side effects, such as death or disability, to their babies, as expressed during the discussion. One woman stated:

“*If I know that there is a problem with that vaccine which they immunize us with, I can’t go back because when I was young, they brought a polio vaccine which they injected many children with, most of whom later became disabled, some even died. So, now if I hear such about the vaccine, I will not come back for another dose*.”

(FGD_Mother_F3 RRH)

For some individuals, the quality and detail of the information they received influenced their decision-making, allowing them to make informed choices. A few participants mentioned they would consider being vaccinated if it could help prevent a serious disease, even if there were some side effects they could tolerate. This highlights their reliance on the credibility and relevance of the information provided. One participant stated,

“*It depends on the adverse events they tell me about. If they inform me that the adverse event is minor and will not affect my baby or my life, I can go ahead with the vaccination. However, if I believe it might impact my baby, then I cannot accept it*.”

(FGD_Mother_F3 RRH)

### 3.6. Experience and Barriers to Reporting Adverse Events

Barriers to the reporting of AEFIs include insufficient information regarding the reporting process, a lack of concern regarding anticipated side effects among healthcare providers and patients, ineffective interactions between women and healthcare personnel, and the influence of alternative community practices.

#### 3.6.1. Lack of Adequate Information

A notable lack of awareness concerning which incidents necessitate reporting, as well as a general unfamiliarity with reporting mechanisms beyond simply notifying health facilities. A participant noted,

“*I am uncertain about the appropriate course of action, as often when we return to the nurses who administered the vaccines, they inform us that it is normal, leading us to endure the side effects until they resolve. However, there are instances where the effects persist*.”

(FGD_Mother_F2 NRH)

#### 3.6.2. Normalization and Endurance of AEFIs (Emergent Theme)

Women reported that they often refrained from reporting adverse effects associated with vaccination, viewing these reactions as normal occurrences and not serious. Common symptoms such as pain at the injection site and fever were frequently downplayed. Additionally, numerous participants reported that healthcare providers reassured them that these side effects were typical and did not require medical attention.

“*Yes, I experienced arm pain for a short period of time. I thought side effects were normal after the injections. I didn’t know we were supposed to report or tell anyone about it unless it became severe*.”

(FGD_Mother_F1 RRH)

“*Often, when you try to go back to inform them, they tend to ignore you. Instead, they keep saying that, “it is normal*.”

(FGD_Mother_F2 NRH)

#### 3.6.3. Trust and Communication Dynamics in Healthcare Interactions (Emergent Theme)

Unfavorable interactions between patients and health workers emerged as a significant barrier to reporting maternal-related side effects. Poor communication or perceived rudeness discouraged reporting, and many mothers expressed that their concerns were frequently overlooked. When they communicated their experiences with side effects, health providers often showed little interest in addressing them, leaving women feeling disempowered to question the authority of health workers. One participant explained:

“*When we go back to the health workers and explain to them, some of them are rude, so you also fear telling them what hurts you, you fear disclosing it to them and instead keep quiet*.”

(FGD_Mother_F2 NRH)

On the other hand, some women reported trust in health workers and confidence in vaccine safety. They felt reassured that mild side effects could be managed appropriately and were more willing to accept vaccines. As one participant stated:

“*We don’t know what the vaccine is, but we trust that the health workers would not harm us*.”

(FGD_Mother_F1 RRH)

“*I do not think there is any danger because, when we come here, our lives are in the hands of the health workers. So, I don’t think it can be dangerous to us because they also get pregnant like us, and they use the same drugs, so I don’t think they can use a dangerous drug, yet they will also use it in future*.”

(FGD_Mother_F1 RRH)

These findings indicate that provider–patient interactions shape AEFI reporting: negative behaviors discourage reporting, whereas trust supports vaccine acceptance.

### 3.7. Desire for Greater Involvement in Vaccine Decision-Making

Participants preferred to be actively involved in decisions about vaccination. They stressed the importance of being informed and consulted before vaccines are rolled out, noting that early engagement helps them understand the purpose of new vaccines and affirms the value of their perspectives. While they recognized that vaccine implementation cannot ultimately be prevented, they emphasized the need for prior education and communication:

“*But if they make a vaccine, before they roll it out in people, since we can’t stop them from making vaccines, they should first come and teach us why they have brought this vaccine and what it will be used for. That way, we shall not have any problem with that*.”

(FGD_Mother_F3 RRH)

One of the mothers expressed the need for health providers to demonstrate the seriousness of a medical condition before introducing new vaccines.

“*I think before the government rolls out vaccines, they should first come to the villages to assess our health situation. They need to find out how many women are suffering from illnesses, the prevalence of tetanus, or how many women have died because they refused to be vaccinated against tetanus. They should conduct research and ask us about the health problems or diseases we face before introducing those vaccines*.”

(FGD_Mother_F3 RRH)

An emerging sense of ownership among participants was evident in their desire to be informed and consulted about vaccines, including types, purposes, side effects, and timing. This reflects women’s evolving role in vaccination decisions and their growing agency. Participants asserted their right to transparent information, further illustrating this emerging agency:

“*I think we have a right to know the types of vaccines which they inject the children or us*.”

(FGD_Mother_F3 RRH)

## 4. Discussion

This study reveals significant gaps in maternal vaccine pharmacovigilance in Uganda. Women generally viewed vaccines, particularly tetanus vaccines given routinely in ANC, as protective and beneficial, yet their confidence was undermined when healthcare providers minimized side effects or failed to clearly communicate schedules and risks. These experiences led to the normalization of adverse events and reduced motivation to report them, even when severe reactions such as fever and arm swelling occurred. Although the COVID-19 pandemic increased vaccine hesitancy in other settings, participants did not link their experiences to pandemic-related concerns. This may be due to the low uptake of COVID-19 vaccines in this population and the separate delivery of COVID-19 vaccines from routine maternal immunization. Nonetheless, the pandemic highlights the need to strengthen trust-based communication between health workers and pregnant women to improve confidence in vaccines.

Our findings highlight the social and relational aspects of vaccine safety monitoring. Trust extends beyond health facilities to peer and family networks that validate women’s vaccine experiences. Women’s growing sense of ownership over vaccine information indicates a shift from passive acceptance to active information seeking and participation, revealing opportunities to co-design communication and reporting strategies with end users. They highlighted their right to be informed and involved in vaccine-related decisions, reflecting a desire for participatory engagement not well captured in prior Ugandan studies [34,35].

Knowledge gaps were evident across participants. Many women depended entirely on healthcare providers for timing and scheduling of doses, reflecting a passive role in the vaccination process. Perceptions of vaccine safety often rested on provider reassurance and the absence of information about serious adverse events, reinforcing an assumption that vaccines posed no risk. This reliance on incomplete or overly positive framing underscores how the credibility and detail of information directly influence willingness to vaccinate, echoing evidence from other settings [36,37,38,39,40]. Strengthening primary healthcare communication through simplified visual tools provided to women during ANC, appointment cards showing vaccines and schedules, and community health worker reinforcement could empower women and improve awareness, sustaining confidence and uptake. Such enhanced communication will go a long way to tackle historical challenges that have been expressed by healthcare providers such as work overload and limited time to communicate effectively with patients [29].

Although awareness of common, non-serious adverse events was relatively experienced, women rarely reported them. These events were widely perceived as “normal” and often dismissed by providers, which further discouraged reporting. Normalization appears to have a dual role: it may help women tolerate mild reactions, but it can also inhibit reporting of severe or disruptive symptoms, underscoring the need for supportive provider communication. Literature suggests that underreporting is exacerbated when only severe events are emphasized [28,41], a pattern also observed here. Moreover, participants demonstrated limited knowledge of the broader pregnancy outcomes that should be reported, highlighting weaknesses in Uganda’s current surveillance framework.

Awareness of national reporting mechanisms was minimal, with most women relying on facility-based reporting through healthcare providers. This reliance was compounded by poor communication, a finding that echoes those from other LMIC contexts [42,43,44]. Given that most participants had only secondary education and worked in informal employment, clear, accessible, and user-friendly reporting tools are urgently needed. Options could include simplified digital platforms and community-based systems that extend reporting opportunities beyond clinical encounters. Early communication during antenatal care is critical to reinforce these pathways [34,40,45,46].

Trust and provider–patient interactions emerged as a major barrier to AEFI reporting. Many women described dismissive or rude responses when reporting side effects, leaving them feeling disempowered and undervalued. Such interactions erode trust and deter future engagement. These factors represent missed opportunities for early detection of safety signals and weaken the credibility of immunization programs [47,48]. Interestingly, despite these experiences, women often maintained trust that healthcare providers and the health system would not harm them, revealing a “trust paradox” in which confidence in the system’s intentions coexists with dissatisfaction in interpersonal communication. While this trust may support vaccine uptake, improving provider communication is essential to encourage timely and complete reporting. Training providers in active listening, respectful care, and compassionate communication could substantially improve both the quality and quantity of maternal AEFI reports [49,50]. However, it is essential to recognize that training alone may not be sufficient to effect change. Systemic challenges such as high patient loads, staffing shortages, limited time allocated for counseling, and a lack of feedback mechanisms for submitted reports often contribute to these dismissive interactions and normalize the experience of side effects. Addressing these systemic issues by strengthening institutional support systems, ensuring timely feedback for healthcare providers, and integrating pharmacovigilance responsibilities into routine workflows will significantly improve provider-patient interactions and foster a supportive environment for open communication regarding side effects. We emphasize that our recommendations aim to enhance existing interactions, and their ongoing implementation will require additional support at the system level.

In our study, we observed reliance on community and informal methods, with individuals seeking advice from family and community members and using traditional remedies such as Dodo leaves to manage perceived vaccine reactions. This underscores the coexistence of informal and formal care systems. Community networks serve as support systems and informal surveillance mechanisms, allowing women to share experiences about vaccine effects before seeking formal care [34,51,52,53,54]. Rather than actively diverting women from healthcare facilities, these practices appear to fill gaps left by formal guidance, particularly when women experience reactions they perceive as incapacitating but are communicated by providers as mild or tolerable. To strengthen pharmacovigilance, it is crucial to engage existing care networks rather than neglect them. Incorporating community health workers and sensitized communities into AEFI surveillance could close the gap between informal and formal systems, enhance early detection, improve reporting, and foster trust in the national pharmacovigilance system.

Finally, women demanded greater involvement in vaccine decision-making. They viewed participation as a right and linked consultation with increased confidence and acceptance. This emerging demand for participatory engagement challenges the traditional, top-down nature of maternal vaccination systems, which have historically relied on directive communication with limited dialogue [47,55,56]. Patient-centered approaches, such as pre-vaccination education sessions, could meet this need, creating space for questions and informed choice. Incorporating women’s perspectives into program design has been shown to improve satisfaction, communication, and program effectiveness and aligns with global guidance on patient engagement in healthcare interventions [45,56,57,58]. For Uganda, adopting strategies that are more inclusive will be essential not only to increase vaccine uptake but also to strengthen pharmacovigilance. These lessons have broader relevance across Africa and align with the WHO Immunization Agenda 2030, which emphasizes community engagement and trust as foundations for successful vaccine introduction [59].

### 4.1. Implication for Policy and Practice

Overall, these findings underscore that maternal vaccine uptake and AEFI reporting are influenced by both interpersonal and systemic factors, including trust in healthcare providers, clarity of information, and opportunities for participatory engagement. Women’s expressed desire for advance education, transparent communication, and involvement in vaccine-related decisions aligns closely with CIOMS [56] guidance, which emphasizes patient engagement throughout the lifecycle of medical products from development to safe use. Integrating participatory approaches into maternal immunization programs can strengthen trust, informed decision-making, and reporting behaviors, while addressing systemic barriers such as limited reporting mechanisms and fear of negative provider reactions. By aligning local strategies with global recommendations on patient involvement, immunization programs can improve both vaccine safety monitoring and community trust, ultimately enhancing maternal and child health outcomes.

### 4.2. Translating Findings into Action

**Figure 2:**
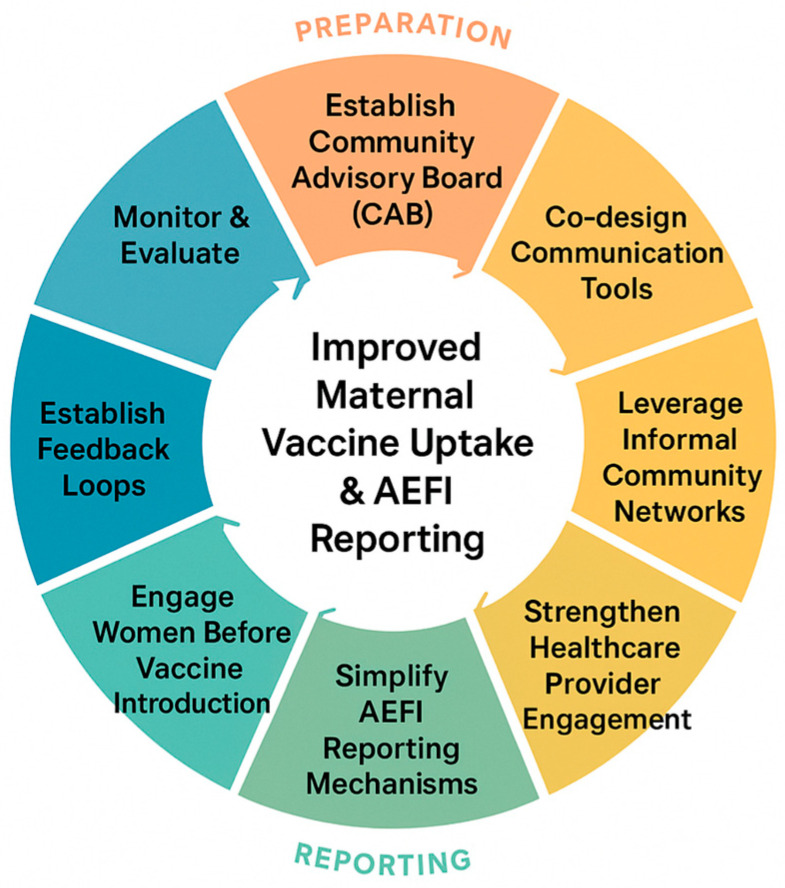
Eight-step adaptable action cycle to strengthen maternal vaccine uptake and AEFI reporting.

To translate our study findings into actionable strategies, we propose a structured adaptable eight-step action cycle for strengthening maternal vaccination and AEFI reporting (Figure 2), while elaborating on each step by outlining the specific actions, target actors and programmatic implication (Appendix A Table A3). This is informed by (1) our findings, including women’s trust in healthcare providers, knowledge gaps, normalization of adverse events, desire for participatory engagement, and reliance on informal care networks; (2) global guidance on patient and community engagement, including CIOMS (2022) [56] and the WHO Immunization Agenda 2030; and (3) evidence from implementation studies demonstrating effective participatory and community-based approaches in LMICs. Each step of the checklist addresses both interpersonal and systemic factors, offering concrete, context-specific actions for program managers and policymakers to strengthen communication, reporting mechanisms, provider engagement, and women’s active participation. By providing a structured, evidence-informed approach, the checklist serves as a practical tool to enhance trust, vaccine uptake, and accurate AEFI reporting, bridging research findings with actionable programmatic strategies.

### 4.3. Strength and Limitation

The primary strength of this study lies in our ability to gather insights from women through group discussions. This approach allowed us to delve into real-world experiences and activities that affect the practical implementation of maternal vaccine safety surveillance. However, the study does have several limitations. Firstly, the small, purposively selected sample reduces generalizability, and restricting recruitment to ANC attendees likely excluded women with less healthcare access. These women who do not utilize formal healthcare may have lower trust in the health system and different perceptions about vaccines and adverse event reporting, potentially biasing our findings toward more positive views of the health system and vaccine programs. Future research should purposively include women outside the formal health system to ensure the perspectives of harder-to-reach groups are represented. Secondly, although focus group discussions (FGDs) facilitated an in-depth exploration of participants’ perspectives, and several strategies were used to reduce social desirability bias including establishing rapport through the use of village heath teams during recruitment, assuring confidentiality, and using neutral prompts some influence may have persisted, particularly when participants discussed their interactions with health workers in relation to reporting of vaccine side effects. Team discussion and cross-group comparison helped to identify and contextualize such patterns during analysis. Additionally, we did not investigate the reporting tools women might prefer or the communication healthcare providers provide regarding vaccines. Although we identified community influence in the data, the study did not explore the perspectives of community influencers and informal caregivers who offer first-line management for perceived reactions. Including these actors, future studies could provide valuable insights into how informal systems interact with and influence formal AE reporting. Lastly, as with qualitative studies, the findings are context-specific and may not be generalized. However, they provide valuable insight into experiences that can inform maternal immunization and pharmacovigilance strategies in Uganda and similar settings.

## 5. Conclusions

This study demonstrates that strengthening maternal pharmacovigilance in Uganda requires attention to both systemic and interpersonal factors, not just technical reporting systems. Vaccine uptake and AEFI reporting are influenced by trust in healthcare providers, clarity of information, normalization of mild adverse events, and opportunities for participatory engagement. Pregnant women should be recognized as partners in vaccine safety monitoring, rather than passive recipients of care.

To achieve this, maternal immunization programs should

1. Provide clear, balanced communication about vaccine benefits and risks, addressing knowledge gaps.
2. Simplify and publicize reporting mechanisms through fit-for-purpose visual tools, digital platforms, and integration with community health workers and informal care networks, enabling broader and earlier reporting.
3. Strengthen healthcare provider capacity through training in respectful, responsive communication, while simultaneously addressing broader systemic challenges such as high patient loads, staffing shortages, limited counseling time, and lack of feedback mechanisms for submitted reports.
4. Engage women before the introduction of new vaccines, incorporating their perspectives into program design and policy decisions to build confidence, ownership, and trust.

Embedding these strategies will enhance trust, informed decision-making, accurate AEFI reporting, and vaccine uptake, while creating a more resilient and responsive maternal pharmacovigilance system.

## Data Availability

The data in this study are available upon request from the corresponding author, due to privacy concerns for the participants involved.

## Supplementary Materials

The following supporting information can be downloaded at: https://www.mdpi.com/article/10.3390/vaccines13121210/s1, Supplementary Material S1: Interview guide.

## Abbreviations

The following abbreviations are used in this manuscript:

AEFI	Adverse Event Following Immunization
ANC	Antenatal Care
CAB	Community Advisory Board
CHW	Community Health Work
EPI	Expanded Programme for Immunisation
FGD	Focus group discussion
GBS	Group B Streptococcus
HCIII	Health Centre III
HCP	Healthcare Provider
LMIC	Low and middle income countries
MOH	Ministry of Health
NMRA	National Medicine Regulatory Authority
NPC	National Pharmacovigilance Centre
NRH	National Referral Hospital
PV	Pharmacovigilance
RRH	Regional Referral Hospital
RSV	Respiratory Syncytial virus
USSD	Unstructured Supplementary Service Data

## Appendix A

**Table A1:** Characteristics of participants involved in FGD.

Characteristics	Iganga Hospital (N = 8) F1	Kawempe NRRH (N = 8) F2	Mbarara RRH (N = 8) F3	Namulonge HCIII (N = 8) F4	MUJHU (Research Centre (N = 8) F5	Total (N = 40)
**Age**						
18–24	03	02	02	06	-	13
25–29	02	05	04	-	01	12
30–39	02	01	02	02	07	14
40–49	01	00	00	00	00	01
**Occupation**						
Informal employment(Hair dressing, roadside stalls, bar attendants, tailoring)	07	06	08	04	02	27
Formal employment	01	02	00	01	00	04
Unemployed	-	-	-	03	06	09
**Marital status**						
Single	-	-	-	01	-	01
Married	08	06	07	07	08	36
Cohabiting	-	02	01	-	-	03
Widow	-	-	-	-	-	
**Education level**						
None	-	-	-	-	-	
Primary	02	02	-	01	07	12
Secondary	04	05	07	07	01	24
Tertiary	02	01	01	-	-	04
**Number of children**						
0–4	07	07	08	08	08	38
5+	01	01	-	-	-	02

**Table A2:** Emerging themes and respondent quotations.

Theme	Subthemes	Quote	Analytical Commentary
Perceptions of Vaccination in Pregnancy	Knowledge about vaccines—importance of vaccines, the common vaccines used, and information about schedules	“*Immunization is very important because, suppose a woman comes for delivery and gets a tear, and they stitch her. If she has been immunized against Tetanus, the wound would not rot, and it would heal easily. But if she has not been immunized, then she takes longer to recover.*” (FGD_Mother_F2 NRRH) “*It helps us in some cases. You can deliver from a dirty place, but if you were vaccinated against tetanus, it helps you not to contract tetanus.*” (FGD_Mother_F1 RRH) “*Immunization is beneficial, but we don’t know when we are supposed to get it or how many times.*” (FGD_Mother_F2 NRH) “*For us, we know that it’s immunization against Tetanus.*” (FGD_Mother_F2 NRH) “*They have always immunized us against tetanus.*” (FGD_Mother_F3 RRH) “*Sometimes we come for immunization and we get doses we don’t know… However, to me I think we have a right to know the types of vaccines that are being injected into the children or us.*” (FGD_Mother_F1 RRH) “*We don’t know the right time when we are supposed to receive the Tetanus injection, but anytime you go is when you can receive it.*” (FGD_Mother_F2 NRH)	Participants understood the general importance of vaccination, particularly tetanus, which is routinely used in antenatal care, but had gaps in knowledge regarding schedules and vaccine purposes. A positive attitude about the vaccines however may not always translate into complete or timely uptake, compromising both individual and public health benefits.
	Prior knowledge and experience influencing vaccine uptake	“*If I know there is a problem with that vaccine, I won’t go back… most of them later became disabled, some even died.*” (FGD_Mother_F2 NRRH) “*Yes, I am willing to accept another vaccine if the health worker has explained about its benefits.*” (FGD_Mother_F2 NRH) “*When I returned for antenatal care the second time, they told me to get it, but I was afraid because the first time I had a fever, and my arm became heavy. I was alone and had to do household chores. The next time, I dodged it.*” (FGD_Mother_F2 NRH) “*When I am pregnant and am told that the treatment can affect the baby, but it is still necessary to treat me, I will refuse treatment. However, if the treatment is for my child and not me, then I am willing to undergo it.*” (FGD_Mother_F2 NRH) “*I do not think there is any danger because, when we come here, our lives are in the hands of the health workers. So, I don’t think it can be dangerous to us because they also get pregnant like us, and they use the same drugs, so I don’t think they can use a dangerous drug, yet they will also use it in future.*” (FGD_Mother_F1 RRH)	Women’s willingness to accept vaccines is not determined solely by personal knowledge or risk perception, but also by the credibility of information sources and interpersonal trust within their communities. Negative experiences or peer stories can amplify fear and hesitation, eroding confidence in vaccination programs. Conversely, trustworthy and empathetic communication from health workers can counter misinformation, provide reassurance, and reinforce positive attitudes toward maternal vaccines.
	Emerging sense of ownership and right to vaccine information	“*I think we have a right to know the types of vaccines which they inject the children or us.*” (FGD_Mother_F1 RRH)	This emerging participatory stance toward maternal healthcare decisions signals a positive evolution toward participatory maternal healthcare. It may imply that Successful vaccination programs must not only ensure access but also foster trust, dialogue, and respect for women’s right to information and expectation for informed consent.
Knowledge and Awareness of AEFI Reporting Mechanisms	Knowledge about possible side effects	“*Like high temperature, body weakness and the arm got swollen “They immunized me when I was three months pregnant. I was affected because I developed a fever and my arm became swollen to the point where I could not hold anything*.” (FGD_Mother_F1 RRH) “*Yes, I experienced arm pain for a short period.*” (FGD_Mother_F1 RRH) “*I think these vaccines have no problem because all the times I have been immunized with all five doses, I have not seen any major issues.*” (FG_Mother_F3_RRH)	Women could identify common mild reactions but tended to normalize them, limiting formal AEFI reporting.
	Knowledge about reporting channels and platforms	“*I do not know what to do because most times when we go back to the nurses… they say, ‘it is normal.’ So you have to endure the side effects until they disappear.*” (FGD_Mother_F2 NRH) “*If you experience any abnormalities after vaccination, please return to the health facility and seek advice.*” (FGD_Mother_F1 RRH) “*They tell you that if you encounter any issues upon returning home, you should come back to the hospital and explain your situation to the health worker so they can assist you in finding a solution.” (FGD_Mother_F4 HCIII)* “*They told us that if the arm becomes heavy, we should give it time, and it will return to its normal state. Also, when we get home, we should not use that arm for anything. It may feel heavy, but it will return to normal over time*.” (FGD_Mother_F3 RRH)	The awareness that formal reporting mechanisms were minimal and that healthcare provider response often discouraged reporting points to a missed opportunity for early detection and response to vaccine safety concerns.
	Community as a source of guidance in managing AEFIs (emergent)	“*When my arm began to swell and hurt, I asked elders who have given birth before, and they told me I should not use that hand.*” (FGD_Mother_F3 RRH) “*After I was immunized, the site where I was injected became itchy. My sister advised me to put a cold bottle on it.*” (FGD_Mother_F3 RRH)	In the absence of guidance from health workers, women relied on community members for advice. This informal support helped manage mild reactions but could perpetuate misconceptions and reduce formal reporting.
Experiences and Barriers in Reporting AEFIs	Barriers to reporting/provider interactions	“*When we go back to the health workers and explain to them, some of them are rude, so you also fear telling them what hurts you.*” (FGD_Mother_F1 RRH)	Fear of negative provider reactions discourages AEFI reporting, highlighting that health worker attitudes are a key determinant of system responsiveness.
	Normalization and endurance of AEFIs (emergent)	“*Most of the time, even if you experience swelling of the arm and intense pain, sometimes accompanied by fever, they keep saying, ‘it is normal.*” (FGD_Mother_F1 RRH)	There is missed opportunity for pharmacovigilance that can lead to underreporting of AEFIs and deprives the immunization program of valuable data needed to monitor safety trends and detect early warning signals.
	Trust and communication dynamics in healthcare interactions (emergent)	“*We don’t know what the vaccine is, but we trust that the health workers would not harm us.*” (FGD_Mother_F1 RRH) “*I do not think there is any danger because, when we come here, our lives are in the hands of the health workers. So, I don’t think it can be dangerous to us because they also get pregnant like us and they use the same drugs, so I don’t think they can use a dangerous drug, yet they will also use it in future.*” (FGD_Mother_F1 RRH)	Participants’ strong trust in health workers sometimes translated into passive acceptance of vaccination recommendations. This is illustrated by participant statements such as “Health workers would not harm us” and “Our lives are in the hands of the health workers High levels of trust can enhance vaccine uptake and compliance, underscoring the importance of provider credibility in maternal vaccination programs. However, excessive trust may lead to passive acceptance, where women do not make informed health choices. This raises concerns for informed consent and health education; while trust is beneficial for uptake, it should not replace clear communication and shared decision-making.
Involvement in Decision-Making	Desire for inclusion and informed participation	“*Before they roll out vaccines, they should first come and teach us about it and what it will be used for.*” (FGD_Mother_F3 RRH)	Women expressed a desire for education and involvement in vaccine-related decisions. This challenges the traditional top-down delivery model and calls for transparent, people-centered approaches that foster trust, empower women to make informed choices, and strengthen both vaccine uptake and accurate reporting.

Note. FGD, focus group discussion; F1–F3, facilities 1–3; NRH, National Referral Hospital; RRH, Regional Referral Hospital; HC, Health Center.

**Table A3:** Description of the eight steps adaptable in the maternal vaccine uptake and AEFI reporting cycle.

Step	Action	Details/Notes	Potential Challenges/Considerations
1	Establish Maternal Vaccine Community Advisory Board (CAB)-	Include pregnant women, CHWs, local leaders, and health providers. Review new vaccine plans, messaging, and schedules. Note: An option to use existing structures, such as Village Health Teams (VHTs), to perform CAB functions, including pregnant women, CHWs, local leaders, and health providers. Functions include reviewing new vaccine plans, messaging, and schedules. Implementation can be adapted to available resources.	Collaboration of EPI-MOH and NPC, and delineating roles. Training the patient representatives to understand their responsibility Ensuring diverse representation. Financial resources to facilitate activities.
2	Co-design communication tools	Workshops with women to develop visual aids, appointment cards, and simplified reporting guides. Test for comprehension.	Time constraints, resources to develop graphic design, translation and prototyping funds.
3	Leverage informal networks	Train mother-to-mother groups, traditional birth attendants, peer networks to recognize and report AEFIs, and link them to formal reporting systems.	Consider inconsistent messaging. Resources: Training modules, supervision, job aids.
4	Strengthen healthcare provider engagement.	Training in respectful, empathetic communication. Address systemic barriers: staffing, workload, counseling time, and feedback for reports.	Heavy workload; high turnover. Resources: Flexible training, job aids, supervision.
5	Simplify reporting mechanisms	Use visual tools, digital platforms, and integrate reporting into CHW/community networks. Ensure accessibility for women with varying literacy levels.	Resources: USSD, toll-free line, Mobile APPs, and manual forms. Funding of tools, translations, and follow-up of notifications to generate complete reports **Challenges:** perception reporting is for HCPs only, internet connectivity for electronic tools if adopted
6	Engage women before vaccine introduction.	Solicit feedback on rollout strategy, messaging, and reporting tools. Incorporate their input into policy/program design.	**Resources:** CHW mobilization, consultation guides, and small incentives.
7	Establish feedback loops	Share AEFI data, uptake results, and program adjustments with women and community networks to sustain trust and ownership.	Define responsibilities: NRA, EPI-MoH Managing community expectation Collaboration on developing messages for feedback.
8	Monitor & evaluate	Track participation, reporting rates, uptake, and community satisfaction. Use results to improve engagement strategies iteratively.	Challenges: Fragmented data; limited qualitative capacity. Resources: Harmonized indicators, integrated tools, survey budget.
