# Supplementary material for: Pregnant Women and Vaccine Safety in Uganda: Knowledge, Barriers, and Opportunities for Engagement
Source: Vaccines (Basel). 2025 Nov 30;13(12):1210. doi: 10.3390/vaccines13121210 (PMC12737582; doi:10.3390/vaccines13121210)
Supplement: Supplementary file 1 [file vaccines-13-01210-s001.zip › vaccines-3962042-supplementary.pdf]

Interview guide for key informant and Focus group discussion

Study: Landscape assessment to understand maternal Vaccine pharmacovigilance in Uganda.

Objectives:

1. Map stakeholders involved in regulation, policy setting, collection and reporting of adverse events following maternal vaccination and their linkages
2. Understand the existing methods, tools, and information flow for collecting and flow of adverse event following maternal vaccination
3. Mapping of current Electronic Health Record (HER) and registries rollout and how they interface with national drug safety reporting system especially in relation to maternal vaccination
4. To understand the Community views, and perceptions on vaccination and reporting of AE during maternal immunization.

Focus group discussion guide for pregnant women and breast-feeding mothers including those that may be participating in clinical trials studying vaccines

Purpose of the interview: In this interview, we want to understand understanding your views on vaccines administered to you during pregnancy, how you receive information on safety of vaccines and how you report any information that you perceive to be an adverse event following vaccination, and you involvement in decision making regarding vaccines given to you . The finding of this assessment will be used to inform development of robust systems that are able to monitor safety of vaccines used in pregnant women.

Instructions for data collectors/interviewers

A: Start by explaining the study objectives to participants and seek for written informed consent.

B: Record Bio-data:
To record details such as age, education level.

C: Open-ended questions:

1. To start off please tell us if you have ever been vaccinated while pregnant
    - If yes probe which vaccines, do they know them and do they know which gestation period or timing they are supposed to get the vaccines
2. Tell us why you think vaccinating pregnant women is important.
    - Probe for general knowledge and also the importance to them as individuals. We are looking for perception about vaccination
3. You mentioned that you were vaccinated while pregnant, were you informed about what to expect after getting vaccinated?

- The question is seeking to find out the source of safety and efficacy information about the vaccines they get. Are they given information about the vaccines they are given?

4. Do you think vaccines can cause side effects? If yes, how did you get to know about this?
5. Have you ever experienced a side effect after vaccination and what did you do?
6. Are you aware of what to do when you expreince an adverse event that you think may have been caused by the vaccine and have you ever reported it? If you reported the adverse event, whom did you report to and why were you motivated to report?
   a. Find more what form did this take to report? (Oral, written, online, WhatsApp, MoH hotlines, Med Safety App, NDA Regional offices?). –the reporting methods are just a guidance for you to elicit an answer if specific answers are not given

7. In case there was reporting, did you get feedback?
8. Would you be willing to have a vaccine even when you are told there could be a risk to you or the unborn child? If so, what would make you make such a decision?

9. If you were given an opportunity to share your concerns about the safety of a vaccine, would your willingness to take the vaccine change?
   - Probe if they have ever been given a chance to express their views. How does this matter to them?

10. Do you think your views and feelings about getting vaccines and the safety of vaccines, or any medicine, should be taken into consideration before any decision is made regarding vaccines or drugs given to you? Please explain
11. What is your final comment about getting a vaccine when pregnant and the reporting of side effects in case they occur?

Thank you for your responses and active participation.
